# Medical embryology and regenerative medicine: research and applications in clinical practice

**DOI:** 10.3389/fcell.2025.1619036

**Published:** 2025-08-26

**Authors:** Julia Soczyńska, Wiktor Gawełczyk, Patrycja Obrycka, Mateusz Żołyniak, Adrian Muzyka, Krzysztof Majcherczyk, Julia Papierkowska, Sławomir Woźniak

**Affiliations:** ^1^ Wroclaw Medical University, Student Scientific Society Anatomia-Klinika-Nauka, Division of Anatomy, Department of Human Morphology and Embryology, Wroclaw, Poland; ^2^ Student Scientific Group of Heart Diseases, Wroclaw Medical University, Wroclaw, Poland; ^3^ Wroclaw Medical University, Department of Human Morphology and Embryology, Division of Anatomy, Wroclaw, Poland

**Keywords:** embryology, stem cells, clinical practice, organoids, regenerative medicine

## Abstract

Medical embryology, as a discipline focused on the developmental processes of living organisms, constitutes the foundation of regenerative medicine through its close integration with genetics, engineering methodologies, and biotechnology, particularly in the field of stem cell cultivation. Investigating its fundamental pillars, such as epigenetics, biomaterials, and bioreactors, within an interdisciplinary framework, is essential for the advancement of modern precision medicine. A thorough understanding of cellular-level processes is undoubtedly the basis for major scientific breakthroughs. Detailed research on the influence of microenvironmental factors on the future function of stem cells, including artificial modulation of external signals that mimic *in vivo* conditions -such as chemical gradients or specific pathways like Notch and Hedgehog -has enabled effective regulation of cellular behavior. Combined with the potential of biotechnology, these advancements open new perspectives for disease treatment and organ regeneration. Taking this progress a step further, the ability to introduce stem cells into damaged tissues, thereby facilitating the formation of functional structures, has led to the growing interest in organoids -three-dimensional models that replicate key functions of real organs. Organoids are currently applied not only in drug screening but are also gaining increasing attention for their role in cancer therapy research. This technology holds the potential to revolutionize medicine, although significant challenges remain, particularly in standardizing cell culture conditions and achieving adequate vascularization of organoid structures. Many advanced regenerative technologies, such as gene editing and bioprinting, are additionally associated with high costs, logistical limitations, and uncertain outcome predictability. Efforts are underway to translate these therapies into clinical practice and to analyze treatment efficacy under real-world conditions, especially in cases where conventional medical approaches prove insufficient. Solving these challenges would mark a scientific breakthrough comparable to the promising results observed in personalized medicine approaches that significantly improve patients’ quality of life. Inspired by the potential of applying modern technologies within an interdisciplinary context, we undertake a comprehensive literature review exploring the integration of embryology and regenerative medicine. We also encourage reflection by addressing the ethical considerations associated with these developments, balancing moral responsibility with legal frameworks.

## 1 Introduction

Medical embryology, as a scientific discipline dedicated to investigating the mechanisms of organismal development across various levels—from genetic regulation to tissue interactions—constitutes a critical foundation for the advancement of regenerative medicine. Notable technological progress in areas such as *in vitro* blastocyst culture and stem cell-based models of peri-implantation embryos has significantly enhanced our understanding of morphogenetic processes and the spatial organization of cells (Cornwall-Scoones and Zernicka-Goetz, 2021; Rossant and Tam, 2022). The dynamic development of tissue engineering techniques, synthetic biology–understood here primarily as the design and engineering of genetic systems–as well as the application of 3D cultures and biomaterials, has enabled the creation of organoids that recapitulate structural and selected functional characteristics of tissues and organs, although full physiological functionality remains limited (Yi et al., 2021). A particularly important area of study is intercellular communication, which governs cell proliferation, differentiation, and migration. Disruptions in signaling pathways can lead to developmental anomalies and diseases such as cancer (Sonnen and Janda, 2021). The growing understanding of these mechanisms is opening new avenues for the development of advanced therapies, particularly in the context of tissue engineering aimed at treating congenital anomalies, including esophageal atresia (Perin et al., 2017). Detailed knowledge of developmental mechanisms contributes to the advancement of precision medicine, particularly in the application of targeted therapies such as gene and cell therapies, offering new prospects for the handling genetic illnesses, including Stargardt disease (Ghenciu et al., 2024; Wang Y. et al., 2024). Future directions in this field involve therapeutic cloning, which may significantly expand the possibilities of transplantation and the treatment of nerve and tissue injuries (Ayala, 2015). Stem cells play a fundamental role in these processes. Their inherent ability to self-organize and generate organ-like structures positions them as central tools in tissue regeneration (Gritti et al., 2021). Among them, pluripotent stem cells (PSCs)—including embryonic stem cells (ESCs) and induced pluripotent stem cells (iPSCs)—form the basis of cell-based therapies employed in the treatment of incurable diseases and tissue damage (Yamanaka, 2020). Hematopoietic stem cells (HSCs), the most thoroughly characterized stem cell type, have long been applied in transplantation procedures, especially for hematologic malignancies such as leukemia, and continue to represent one of the most used forms of cell therapy (Zakrzewski et al., 2019). This is exemplified by data from 2018, indicating a total of 93,105 hematopoietic cell transplants (HCTs) performed globally, including 44,425 allogeneic and 48,680 autologous transplants (Atsuta et al., 2024). This upward trend has continued, and, according to one of the most recent EBMT reports, 47,731 HCTs were performed in 2023 (42.9% allogeneic, 57.1% autologous), confirming not only a full recovery from the pandemic-related decline but also highlighting ongoing growth—particularly in allogeneic transplants for myeloid malignancies (Passweg et al., 2025). Neural stem cells (NSCs) and mesenchymal stem cells (MSCs) are also under intensive investigation for their therapeutic potential in the treatment of neurodegenerative disorders, such as Alzheimer’s disease (Duncan and Valenzuela, 2017). Advances in embryology have also significantly contributed to the treatment of infertility, particularly through *in vitro* fertilization (IVF), where embryos are cultured under controlled laboratory conditions and evaluated by embryologists based on quality and implantation potential (Greco et al., 2020; Jiang and Bormann, 2023). In the United States alone, 203,119 assisted reproductive technology (ART) procedures were performed in 2018, resulting in 73,831 live births and the delivery of 81,478 infants (Sundream et al., 2018). Moreover, studies on the interactions between gene regulatory networks and morphogenetic events have enhanced our understanding of embryogenesis and enabled the development of therapeutic strategies for conditions such as early pregnancy loss (Shahbazi, 2020). The dynamic transformations and expanding capabilities within the field of embryology—as a cornerstone of regenerative medicine—necessitate a thorough analysis of their interconnections and implications for contemporary clinical practice (Larijani et al., 2017). In this context, we aim to explore the latest scientific achievements in embryology and their practical applications in disease therapy and tissue regeneration. We highlight the close interplay between these domains and its influence on the development of modern medicine. This study presents key signaling pathways and embryological mechanisms, with particular emphasis on the role of the stem cell microenvironment, which significantly affects stem cell function, differentiation, and therapeutic potential. We further discuss organoids as innovative therapeutic tools and provide clinical examples where stem cell-based and embryology-derived technologies have produced promising outcomes. Additionally, we address ethical concerns associated with regenerative therapies, particularly regarding the use of embryos and PSCs, and outline the major challenges, limitations, and future prospects of this rapidly evolving field.

## 2 Embryological pathways and mechanisms in regenerative medicine

Regenerative medicine is an interdisciplinary field that integrates principles of engineering and biological sciences to develop methods for restoring, repairing, and generating tissues and organs. This includes the potential to replace those that have been damaged or affected by pathological processes (Mao and Mooney, 2015). In recent years, its rapid development has been particularly evident in the healthcare sector, where therapeutic approaches such as cell therapy, *in vivo* reprogramming, synthetic biology, as well as antifibrotic and anti-inflammatory therapies have been widely applied (McKinley et al., 2023). Stem cells play a pivotal role in these processes. Owing to their ability to differentiate into various cell types and secrete bioactive substances that influence resident cells through paracrine signaling, stem cells actively contribute to tissue regeneration and repair (Hodgkinson et al., 2016). Their differentiation potential has led to the classification of stem cells into various types such as: unipotent, multipotent, pluripotent (Mahla, 2016). Unipotent stem cells, present in specific tissues, are capable of differentiating into only 1 cell type, such as macrophage precursor cells or skeletal muscle satellite cells (Szabłowska-Gadomska et al., 2017). In contrast, multipotent stem cells, which are tissue-specific, can differentiate into multiple cell types derived from a single germ layer. An example is HSCs, which are capable of generating all types of blood cells. (Dulak, 2020). MSCs, which are classified as multipotent cells, constitute a heterogeneous population capable of differentiating into myocytes, adipocytes, chondroblasts, and osteoblasts, depending on their tissue of origin. As such, they play a crucial role in tissue regeneration and repair by secreting various growth factors and modulating the immune response (Yan et al., 2023).

PSCs are of particular importance in regenerative medicine due to their expression of genes such as *Oct4* and *Nanog*, which enable differentiation into nearly any cell type of the adult organism, excluding placental cells (Kmiecik et al., 2015; Miyamoto et al., 2015). ESCs and iPSCs are typical examples of PSCs (Dulak et al., 2015).

Nonetheless, the effective utilization of stem cells for regenerative purposes necessitates an in-depth understanding of the signaling pathways that modulate their behavior, guide their differentiation, and facilitate their integration into host tissues, as detailed below.

One of the key pathways is the Notch signaling pathway, which regulates cell development, organogenesis, and tissue homeostasis. This pathway functions through interactions between Notch receptors (Notch1–4 in mammals) and their ligands, such as Jagged1 (JAG1), Jagged2 (JAG2), and Delta-like ligands (DLL1, DLL3, DLL4), leading to the activation of target genes (Shi et al., 2024). The Notch signaling pathway can be divided into two types: the canonical and the non-canonical pathway. The canonical pathway activates the expression of genes such as MYC, a key regulator of the G1 to S phase cell cycle transition, and HES1, which is essential for maintaining stem cell self-renewal. In contrast, the non-canonical pathway regulates factors such as NF-κB, involved in inflammatory responses and cell survival, and Rac1, which controls cell motility, shape, and cytoskeletal organization (Katoh and Katoh, 2020). Depending on the cancer type and stage, Notch signaling may act as either an oncogene or a tumor suppressor. Based on this knowledge, modulation of the Notch pathway enables potential therapeutic strategies—for example, in the treatment of small-cell lung cancer (SCLC), where the broadly expressed DLL3 ligand in SCLC cells is targeted using antibodies and specific inhibitors (Zhang et al., 2023). Importantly, this signaling pathway does not act in isolation but closely interacts with growth factors to modulate the behavior of MSCs in their niche, while platelet-derived growth factor (PDGF) secreted by stem cells or resident tissue cells influences both Notch and Wnt pathways to coordinate regeneration (Gerli et al., 2019). Another essential signaling mechanism is the Wnt/β-catenin pathway, a canonical signal transduction cascade. Upon activation by Wnt proteins, β-catenin becomes stabilized and translocates to the nucleus, where it interacts with transcription factors (TCF/LEF) to induce the expression of target genes such as c-Myc and Cyclin D1, which are involved in cell cycle progression from G1 to S phase. This pathway plays a great role in stem cell renewal and organogenesis (Krishnamurthy and Kurzrock, 2018). In addition non-canonical Wnt pathways (such as Wnt/PCP and Wnt/Ca^2+^) also play important roles in tissue regeneration by regulating cell polarity, migration, and cytoskeletal organization (Cai et al., 2018). Similar to the Notch pathway, the Wnt/β-catenin pathway is also implicated in the development of cancer. Aberrant activation of the Wnt/β-catenin pathway-resulting from genetic mutations, epigenetic modifications, or cross-talk with other signaling pathways-can lead to uncontrolled cell proliferation, epithelial-to-mesenchymal transition (EMT), and the maintenance of cancer stem cells (CSCs). These processes contribute to therapy resistance, enhanced migratory capacity, and increased metastatic potential (Song et al., 2024). However, understanding the underlying mechanisms of this pathway opens new avenues for innovative cancer therapies. Its hyperactivation can induce apoptosis in tumor cells, while inhibition of the pathway may arrest the cell cycle and promote differentiation of oncogenic cells (Trejo-Solis et al., 2021). Additionally, the Hedgehog (Hh) signaling pathway is of particular interest due to its essential role in organogenesis, tissue regeneration, and homeostasis across nearly all organs. It facilitates intercellular communication during embryonic development, and its dysregulation is associated with the development of various cancer types (Carballo et al., 2018). In mammals, the Hh gene family consists of three members: *Desert hedgehog* (DHH), *Indian hedgehog* (IHH), and *Sonic hedgehog* (SHH)—the latter being widely expressed in human tissues and involved in the development of the brain, spinal cord, skin, hair, teeth, and lungs (Yang et al., 2021). Although the Hh pathway is generally inactive in adults, it may be reactivated in specific conditions such as wound healing, where it plays a crucial role in maintaining somatic and PSCs important for tissue repair. This includes mammary, skin, neural, erythropoietic, pulmonary, and certain epithelial cells of internal organs. Consequently, the pathway is essential for the regeneration of the lung epithelium, prostate epithelium, and exocrine pancreas cells (Skoda et al., 2018).

Importantly, growth factors complement the actions of stem cells and signaling pathways by playing a vital role in embryological and regenerative processes. They bind to specific receptors and activate intracellular signaling pathways that trigger key cellular events such as migration, survival, adhesion, proliferation, growth, and differentiation (Mitchell et al., 2016). Notably, both stem cells and tissue-specific cells contribute to these mechanisms through autocrine and paracrine signaling involving not only growth factors but also cytokines. This promotes the recruitment, proliferation, and differentiation of cells, extracellular matrix (ECM) synthesis, and tissue remodeling, especially in areas requiring repair, such as the damaged rotator cuff (Prabhath et al., 2018). Among this group of proteins, several key growth factor families can be distinguished, including: PDGF, vascular endothelial growth factor (VEGF), epidermal growth factor (EGF), fibroblast growth factor (FGF), insulin-like growth factor (IGF), and hepatocyte growth factor (HGF) (Tarvestad-Laise and Ceresa, 2023). PDGF, a polypeptide growth factor, plays a fundamental role in cell proliferation, differentiation, and migration, as well as in collagen synthesis and the transformation of hepatic stellate cells into myofibroblasts—making it a central factor in the pathogenesis of liver fibrosis. It is composed of four subunits (PDGF-A, -B, -C, -D), which form five dimeric isoforms (PDGF-AA, -BB, -AB, -CC, -DD) and act through PDGFR-α and PDGFR-β receptors. These interactions activate several downstream signaling pathways—including Ras/MEK/ERK, PI3K/Akt, PLCγ, and JAK/STAT—that regulate cell proliferation and differentiation, survival and growth, protein activation, immune responses, and gene transcription (Ying et al., 2017). VEGF is a key pro-angiogenic factor that promotes endothelial cell proliferation, migration, and survival, enhances vascular permeability, and plays a pivotal role in both physiological (e.g., wound healing, placental development) and pathological (e.g., cancer, diabetic retinopathy) angiogenesis. The VEGF family includes VEGF-A (with multiple isoforms), VEGF-B, VEGF-C, VEGF-D, VEGF-E (virus-derived), VEGF-F (snake venom-derived), and placental growth factor (PlGF). These factors bind to VEGFR-1, VEGFR-2, and VEGFR-3, with VEGFR-2 exhibiting the most potent pro-angiogenic activity. Neuropilins (NP-1, NP-2) function as co-receptors that modulate VEGFR signaling (Stanca Melincovici et al., 2018). EGF initiates a tyrosine kinase receptor signaling cascade that regulates cell proliferation, differentiation, and survival. All EGFR ligands are assembled as membrane-bound precursors and released following proteolytic cleavage. EGF plays a significant role in tissue homeostasis and is involved in regenerative and repair processes, for example, in the kidneys and skin (Singh et al., 2016). FGFs are a family of proteins involved in fundamental biological processes, including embryonic development, angiogenesis, tissue homeostasis, wound healing, and tumorigenesis. They act through the activation of tyrosine kinase FGFR receptors and intracellular signaling pathways (Giacomini et al., 2021). IGF plays a crucial role in cell proliferation, apoptosis inhibition, and organismal growth and development. It also contributes to the maintenance of bone and muscle mass, making it a focus of research into life extension and improving health during aging (Osher and Macaulay, 2019). HGF is a key promoter of liver regeneration. It regulates cell growth, migration, and morphogenesis through interaction with the c-Met receptor and activation of JAK/STAT3, PI3K/Akt/NF-κB, and Ras/Raf signaling pathways. These mechanisms are of particular importance in regenerative therapies for liver diseases, including fibrosis treatment, hepatocyte regeneration following inflammation, and post-transplant recovery (Zhao et al., 2022).

**FIGURE 1:**
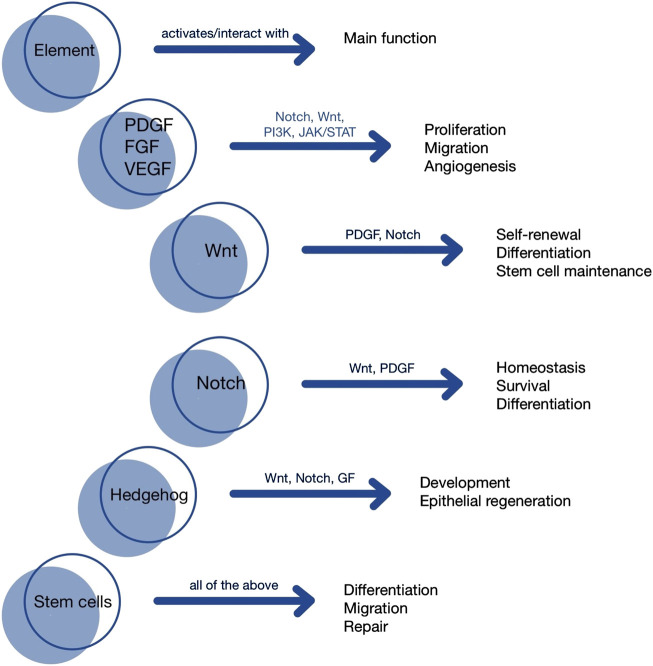
Visualization of signaling pathways].

In summary, the regenerative capacity of stem cells relies not only on their intrinsic properties but also on the complex interplay of signaling pathways and growth factors that modulate their behavior within the tissue-specific microenvironment. Data are presented both in Table 1, and Figure 1.

**TABLE 1:** Summary of the described pathways.

Trail	Physiological role	Role in the pathogenesis of tumor	Therapeutic possibilities
Notch	It regulates cell development, organogenesis, and tissue homeostasis. Its action is mediated through the interaction of Notch receptors (Notch1–4) with their ligands (JAG1, JAG2, DLL1, DLL3, DLL4)	“It may exhibit both oncogenic and tumor-suppressive roles, depending on the cancer type and stage	Modulation of the Notch pathway enables cancer treatment, for example, by targeting the DLL3 ligand in small-cell lung cancer (SCLC) using inhibitors and antibodies
Hedgehog (Hh)	It regulates organogenesis, regeneration, and organ homeostasis. This pathway is activated, among other contexts, during wound healing and tissue regeneration	Aberrant activation of the Hedgehog (Hh) signaling pathway leads to pathological proliferation of cancer cells	Potential applications in the regeneration of pulmonary, prostatic, and pancreatic epithelium. Ongoing research is focused on targeting this pathway for cancer treatment
Wnt/beta-katenine	Stabilization of β-catenin and its translocation to the cell nucleus, where it activates genes associated with stem cell renewal and organogenesis	“Excessive activation leads to tumorigenesis, epithelial-to-mesenchymal transition, and treatment resistance. It plays a pivotal role in cancer progression and the maintenance of cancer stem cells	Potential therapies based on either inhibition or hyperactivation of the pathway, aimed at inducing apoptosis or causing cell cycle arrest in cancer cells

## 3 The role of the stem cell niche

### 3.1 Fundamental components of the niche

Stem cells, which represent a fundamental pillar of modern regenerative medicine, require specific conditions for successful differentiation. They include the presence of growth factors, cytokines, enzymes, and components of the ECM (e.g., collagen), ions, as well as mechanical stimuli. This encompasses physical parameters such as stiffness, which is considered a critical determinant, and viscosity (Pennings et al., 2018; Zhou et al., 2019; Lim et al., 2022). Blood and lymphatic vessels, nerves, and elements of the immune system further complement this framework, collectively forming the stem cell niche. These cells are extremely sensitive to their surrounding environment; therefore, any alteration—even those associated with physiological aging—can directly impact their behavior. Communication between stem cells and their niches determines their ability to maintain homeostasis and respond to tissue demands (Fuchs and Blau, 2020; Farahzadi et al., 2023). This reciprocal interaction is exemplified in MSCs, which modulate macrophage activity through the secretion of soluble mediators. These signals shift macrophage polarization from the pro-inflammatory M1 phenotype to the anti-inflammatory M2 subtype. Furthermore, interleukin 17 (IL-17), whose production is stimulated by MSC, enhances the phagocytic capacity and effector functions of neutrophils (Hsu et al., 2013). Depending on external stimuli—such as insulin-like growth factor 1 (IGF-1) — MSC can be directed towards specific differentiation pathways. It has been demonstrated, for example, that IGF-1 induces osteogenic differentiation and delays the commitment of placental mesenchymal stem cells (PMSCs) towards the myogenic lineage, in contrast to IGF-2, which promotes the development of myoblast cells (Aboalola and Han, 2017; Liu et al., 2022). Recent reports have demonstrated that bone marrow-derived stem cells (BMSCs) placed within a neuronal niche can undergo transdifferentiation into neuronal cells. This plasticity, driven by the surrounding niche, is also being leveraged *in vitro* to alter culture conditions—such as those used for hepatic cells—to enable their transdifferentiation into insulin-producing pancreatic islet-like cells (Elsayed Azab et al., 2021). We draw attention to recent reports that have confirmed the existence of a reprogramming process through which the endothelium of major embryonic arteries contributes to the formation of HSCs. This finding has revolutionized the previously accepted yolk sac–based model of hematopoietic origin (Dzierzak and Bigas, 2024). The newly generated HSCs remain highly responsive to niche-derived signals, and an improved understanding of these interactions paves the way for the refinement of *ex vivo* cell culture protocols (Cain et al., 2024). Furthermore, these discoveries hold the potential to inform novel strategies in regenerative medicine. There is now evidence supporting the role of the human placenta as an extraembryonic niche facilitating the development of HSC. The presence of CD34^+^ HSCs has been detected throughout the entire duration of pregnancy. Expression of CD117 and CD41 is observed during the first and second trimesters, with a peak occurring in the second trimester. Stromal cells present within the placental microenvironment exhibit the capacity to support hematopoiesis and maintain the stem cell population. This makes the placental stroma a promising tool for therapies targeting bone marrow damage and related hematological deficiencies (Gao et al., 2018; Jovicic et al., 2025). Despite the promising proliferative potential and satisfactory availability of placental cells, obtaining pure cultures devoid of maternal cell contamination remains challenging. A key obstacle is the overgrowth of maternal-derived MSC in the established cell cultures (Sardesai et al., 2017). Although stem cell niches across different organs share common structural and functional components, they exhibit significant heterogeneity depending on their tissue-specific localization. These differences manifest in the way they generate signaling pathways and respond to physical and biological stimuli, thereby influencing the functional state of the resident stem cells (Singh et al., 2019). Given these abilities, stem cells have been implemented in sophisticated therapeutic techniques. For instance, HSCs are utilized in bone marrow transplantation to regenerate blood cells in patients with hematological malignancies. Similarly, stem cells derived from the skin are employed in regenerative therapies for patients suffering from extensive burns (Cable et al., 2020). For example, blood and intestinal epithelium are characterized by high rates of proliferative activity, in contrast to the relatively quiescent nervous tissue. Positioned between these extremes, skeletal muscle tissue exhibits substantial regenerative potential, which, however, is activated only under specific conditions (Mannino et al., 2022). Throughout the lifespan of a niche, its architecture undergoes modifications not only in response to pathological conditions but also as part of natural maturation processes (Hicks and Pyle, 2023).

### 3.2 Clinical significance

Proper functioning of the niche is associated with the desirable regulation of stem cells and maintenance of homeostasis. Its aging is linked to the disruption of normal stem cell processes, which consequently contributes to the development of various diseases (Farahzadi et al., 2023). Changes in molecular-level determinants play a crucial role in this context. The affected stem cells include, among others, hematopoietic, mesenchymal, and NSCs, which will be the focus of our attention (Pérez et al., 2018).

The embryonic period marks the beginning of niche formation. During this time, NSCs within the ventricular zone of the neural tube initiate the formation of all central nervous system structures, including neurons and glial cells. Interestingly, certain populations of these stem cells can remain quiescent from embryogenesis and only become activated in adulthood. Notable adult neurogenic niches such as the subventricular zone adjacent to the lateral ventricles and the subgranular zone of the dentate gyrus are enriched in regulatory factors—such as doublecortin—and their dysfunction has been implicated in neurodegenerative diseases like Parkinson’s and Alzheimer’s (Llorente et al., 2022). Damage to these niches often results in the depletion of resident NSCs. Importantly, biological sex influences neurogenic niche dynamics; for instance, menopause in females is associated with a reduction in stem cell populations, which may contribute to gender differences in disease susceptibility (Velikic et al., 2024). The stem cell niche plays a crucial role also in the context of the progression of cancer cell processes (Wagers, 2012). Certain pediatric leukemias have been linked to the persistence of fetal-like niches, with origins traced back to prenatal development. This supports the theory that abnormal niche formation and maturation could underlie developmental origins of disease (Hicks and Pyle, 2023). In the context of cancer, niches also serve as sanctuaries for CSCs, protecting them from immune surveillance. Metastatic colonization, such as in the liver, often involves niche remodeling by CSCs, for instance via inhibition of proteases and upregulation of Tissue Inhibitor of Metalloproteinases 1 (TIMP-1), which aids in the establishment of pre-metastatic niches. Given the dependence of CSCs on supportive niches, niche-targeted strategies—such as oxidative modulation—represent promising therapeutic avenues that can be tailored to tumor type and stage (Plaks et al., 2015; Oshimori et al., 2021; Ju et al., 2022).

### 3.3 Niche engineering

Therapeutic potential continues to expand rapidly, reinforcing optimism for future breakthroughs. The ability of niches to condition stem cell responses—for instance in injury—provides a foundation for controlled repair processes. Leveraging this knowledge, advanced technologies now enable the development of *in vitro* niche models and the genetic editing of both stem cells and their niches (Chacón-Martínez et al., 2018). In replacement therapies, human PSCs, especially ESCs, are of particular relevance due to their relatively easy maintenance in culture and capacity to differentiate into mesoderm, endoderm, and ectoderm lineages (Martinez et al., 2022; Tian et al., 2023). Their responsiveness to hormetic stimuli further underscores the influence of the niche (Calabrese, 2022). Nevertheless, challenges remain in optimizing the delivery of regulatory molecules and components into niches to promote targeted stem cell differentiation and function (Subramanian et al., 2024). Strategies to overcome transplantation barriers include the use of biomaterials that mimic the microenvironment. Depending on their physical properties, such scaffolds not only provide structural support but also emit biochemical signals that influence cellular differentiation (Zhao et al., 2021). Modifying the surrounding conditions of stem cells—such as ionic signals released from engineered inorganic crystalline materials—can trigger niche-specific responses, although clinical outcomes of such approaches are still under investigation (Zhou et al., 2019). There are reports describing composites of bioceramics and hydrogels that, through the release of lithium, magnesium, and silicon ions, contribute to the specialization of bone marrow-derived cells. In contrast, strontium and silicon ions have been associated with the promotion of angiogenesis, particularly in relation to human umbilical vein endothelial cells (Zhang et al., 2025). Recent scientific advances have enabled the reconstruction of stem cell niche environments, allowing precise *in situ* assessments. Laboratory modeling requires not only the presence of stromal cells and structural protection but also a balanced scaffold that supports the desired microenvironment (Abdul-Al et al., 2021). These scaffolds can be integrated with biological materials that transduce exogenous signals (e.g., light, electric fields, ultrasound, or magnetic forces) into cellular instructions, simulating the dynamic interactions found in living tissues (Gelmi and Schutt, 2021). Hydrogels—polymer networks with a three-dimensional structure—are widely used biomaterials, applied, for example, in the treatment of osteoarthritis. A notable example is the study by Park Yong-Beom et al., which involved seven patients treated with allogeneic human umbilical cord blood-derived mesenchymal stem cells (hUCB-MSCs) suspended in a hyaluronic acid hydrogel. The study demonstrated the presence of repair tissue after 12 weeks, with its maturation and durability confirmed by magnetic resonance imaging after 3 years. There is growing evidence that preconditioning of MSCs prior to transplantation enhances their clinical potential by supporting large-scale expansion and functional optimization. These cells can be modulated to preserve their phenotype or directed towards specific cell lineages (Park et al., 2017; Madl and Heilshorn, 2018; Sun et al., 2023). 3D bioprinting is gaining traction as an alternative to hydrogel-based methods, enabling scaffold fabrication followed by cell seeding. In this context, porosity and biocompatibility are critical parameters. These technologies have been applied in cartilage repair and other regenerative models (Huang J. et al., 2021; Su et al., 2021). More recently, the development of three-dimensional bacterial cellulose–graphene foam has been shown to support neuronal stem cell growth and differentiation, offering a promising approach for neurodegenerative diseases (Guo et al., 2021). To mitigate immune responses, scaffold-free strategies such as cell sheets and spheroids are being explored. Cell sheets can be harvested using stimuli such as temperature, light, or magnetic fields in combination with titanium dioxide, while spheroids are formed through cellular aggregation. Despite their promise, challenges remain, particularly regarding efficacy and the need for standardized protocols in stem cell manipulation (Liu et al., 2024).

## 4 Organoids

Organoids are three-dimensional (3D) clusters of cells cultured outside the body in a controlled environment. These cellular structures possess the ability to self-organize and differentiate into specialized cell types, closely mimicking the early stages of embryonic development (Qian et al., 2019; Corrò et al., 2020). Organoid platforms employ two stem cell sources: pluripotent cells (ESCs/iPSCs), which can develop into many different cell types, and more lineage restricted adult stem cells (ASCs) (Dutta et al., 2025). The control of the microenvironment is crucial for the synthesis of organoids. The use of hydrogels such as Matrigel, replicate the ECM to promote proliferation and differentiation into the specific cell type (Gan et al., 2023). Organoids provide a useful approach for investigating organ development, disease mechanisms, and drug effects. These topics are challenging or impractical to study directly in humans (Ge et al., 2024).

### 4.1 Organoids in scientific research

Organoids allow researchers to study disease modelling, offering a hopeful, ethical alternative to animal models (Ho et al., 2018). For instance, brain organoids hold significant promise for studying neurodegenerative diseases including, Alzheimer’s and Parkinson’s diseases (Urrestizala-Arenaza et al., 2024). Research demonstrates that brain organoids can replicate pathological hallmarks of Alzheimer’s disease, such as the presence of phosphorylated tau protein, the aggregation of beta-amyloid, and the role of nitric oxide release in neuronal damage (Papaspyropoulos et al., 2020). Studies conducted on chimeric human cerebral organoids (chCOs) have shown that the presence of the apolipoprotein E4 (APOE4) genotype correlates with increased cholesterol accumulation in neurons and elevated levels of phosphorylated tau protein (Huang et al., 2022).

The ability of retinal organoids to replicate the structure and function of the retina allows for a more physiological studies of disease mechanisms and drug effectiveness compared to conventional 2D models (Mousavi et al., 2025). Similarly, midbrain organoids are valuable for modeling Parkinson’s disease. Midbrain organoids contain dopaminergic neurons and other cell types found in the human midbrain, such as astrocytes (Solana-Manrique et al., 2025).

### 4.2 Organoids in personalized therapies

After analysing the use of organoids in scientific research, we will now focus on their application in personalized medicine. The role of tumour organoids in personalized medicine presents considerable potential. Samples collected from tumour biopsies can be used to develop patient-derived organoids (PDOs). These organoids enable clinicians to test drug efficacy and select optimal therapies for individual patients (Chen et al., 2024). Cancer organoids are also used to evaluate radiotherapy responses. However, organoids require further investigation to establish their role as reliable components of personalized anticancer treatment strategies (Kretzschmar, 2021). Another example of the application of organoids are the treatment of cystic fibrosis (CF), a genetic disorder caused by mutations in the *CFTR* (CF transmembrane conductance regulator) gene. These mutations impair the function of chloride channels, leading to the accumulation of viscous mucus in multiple organ systems, including the respiratory and digestive tracts (Dickinson and Collaco, 2021; Jia and Taylor-Cousar, 2022). Current CF treatment strategies rely on symptomatic management and CFTR modulator therapy, which increase the number of functional channels and restore their activity. However, patients with rare *CFTR* mutations cannot benefit from this approach due to the inability to conduct clinical trials for such a limited population (Conti et al., 2025). The capability of patient-derived intestinal organoids to identify genetic variants of CFTR mutations allow the potential application of modulators. These modulators could be used for rare variants that were previously untreated with these substances. This capability enables personalized evaluation of therapeutic efficacy and the determination of optimal treatment (Conti et al., 2022).

### 4.3 Organoids in regenerative medicine

**FIGURE 2:**
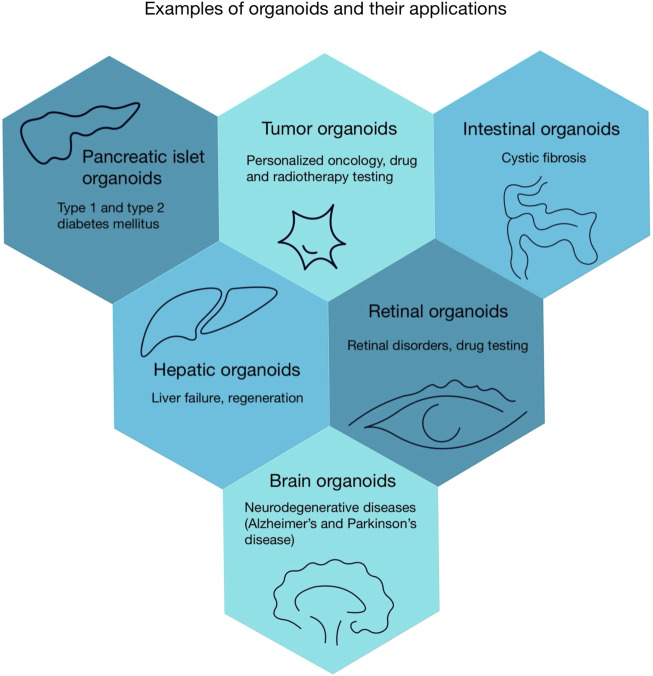
Examples of organoids and their applications.

After discussing how organoids contribute to the development of personalized therapies, we will now proceed to consider their application in regenerative medicine. Organoids offer new opportunities in regenerative medicine. For instance, liver organoids could provide an alternative to liver transplantation. Transplantation is currently the only effective treatment for liver failure, but requiring lifelong immunosuppression (Olgasi et al., 2020). Studies in animal models demonstrate that transplanted hepatocellular organoids successfully engraft and demonstrate functionality. This is evidenced by the detection of human liver markers, such as human albumin, in recipient models (Jalan-Sakrikar et al., 2022). The ability of organoids to generate safe, functional biological structures positions them as a transformative tools for regenerative therapies (Gu et al., 2023). Another notable example is research focused on generating insulin-secreting islet organoids from stem cells, which may represent an advancement in diabetes therapy (Yu et al., 2024). Despite the morphological and functional similarities between pancreatic organoids and native pancreatic beta cells, these structures have limitations. They exhibit impaired responsiveness to elevated blood sugar level and lack of some signature biomarkers characteristic of mature pancreatic beta cells (Yang et al., 2024). The various organoids and their uses are illustrated in Figure 2.

### 4.4 Brain organoids

After discussing the various organoid types, we will now proceed to a detailed analysis of brain organoids. Over the past few years, brain organoids have become more important in neurological research. In brain organoid development, dual inhibition of SMAD signalling (Suppressor of Mothers against Decapentaplegic) is essential for inducing stem cells to differentiate into neuroectodermal tissue (Mulder et al., 2023). Creating organoids specific to different brain regions requires modulating signalling pathways such as WNT, SHH, FGF and BMP. This approach enables the modelling of diseases in various brain areas (Susaimanickam et al., 2022). To evaluate brain organoids, it is necessary to apply various techniques to assess the function and structure of the neuronal cells. Techniques like immunohistochemistry allow for the identification of markers and analysis of cellular organization within the organoid (Bell et al., 2025) Another example involves employing Transmission Electron Microscopy (TEM), which reveals neuronal connections and other neuronal structures (Noh et al., 2025). A key limitation in the development of brain organoids is their inability to replicate the human brain’s complexity due to the absence of critical cell types (e.g., immune cells) and the inability to mimic intricate microenvironmental signals (Harati and Wang, 2024).

### 4.5 Challenges and Limitations

Despite their potential, organoids face significant limitations. Currently, the size and proportions of organoids pose challenges for their future application in regenerative medicine. One of the main factors inhibiting their growth is the lack of vascularization. This limitation restricts gas exchange, the removal of metabolic waste products, and the delivery of essential nutrients. (Huang et al., 2021). As a result, the cultured tissues gradually undergo necrosis before reaching several hundred micrometres in size (Zhang et al., 2021b; Andrews and Kriegstein, 2022). Moreover, cultured organoids differ from original tissues. For example, analyses of kidney organoids revealed that up to 20% of cells are unrelated to kidneys (Romero-Guevara et al., 2020). Another challenge for scientists is finding an alternative to Matrigel, which is currently widely used. Matrigel is characterized by its high cost and variability between batches, which hinders the development of organoids (Sulaksono et al., 2024). Studies highlight key limitations that require further research to advance this technology. Overcoming these obstacles will pave the way for new era in patient care.

### 4.6 Emerging Workarounds

In response to these limitations, new solutions are emerging. One of these solutions uses microfluidic perfusion to monitor the microenvironment through precise regulation of fluid and nutrient supply (Saorin et al., 2023). Studies shows that certain perfusion systems incorporating microvessel printing reduce the risk of necrosis in large tissue models (Grebenyuk et al., 2023). To solve vascularization problems, organoids can be co-cultured with endothelial cells, which leads to the formation of vascular networks and improves oxygen and nutrient delivery (Zhao and Haddad, 2024).

### 4.7 Standardization and reproducibility

Organoid research represents a breakthrough in regenerative medicine and disease modeling. However, researchers face many challenges. One of the foremost challenges in organoid development is standardizing culture conditions and ensuring reproducibility.

The heterogeneity of tissue sources, processing methods, and culture-medium components leads to differences in organoid culture outcomes (Guo et al., 2024). A prime example is the use of Matrigel, which is derived from mouse sarcoma. This leads to batch-to-batch variability that negatively impacts culture reproducibility. Finding an alternative organoid matrix that offers stable physical parameters for organoid cultivation is crucial (Huang et al., 2025). Researchers are currently developing synthetic alternatives to ECM such as nanomaterial hydrogels. It is important to guarantee their functionality, stability, and safety (Kim and Cha, 2025). The implementation of standardized guidelines is expected to promote the further practical development of organoids (Lee et al., 2024). To ensure comparability of organoid research results, new initiatives have been introduced. One of the initiatives is the Human Endoderm-Derived Organoid Cell Atlas (HEOCA), an atlas compiling data from organoid cultures that contains approximately one million cells. This resource will enable researchers to more easily compare organoids and develop new culture protocols in the future (Xu et al., 2025). Another project is the Organoid Cell Atlas, part of the Human Cell Atlas initiative. This effort is focused on standardizing cultivation methods, analysing data, and accelerating organoid research to advance this technology (Bock et al., 2020). In conclusion, key priorities include harmonizing tissue origins, standardizing growth media, establishing alternative organoid matrices, and improving global collaboration on research outcomes.

## 5 Overview of clinical case studies

### 5.1 Stem cell therapy

Stem cell therapy is one of the most promising and forward-looking fields in medicine. This therapy offers a vast array of possibilities for patients suffering from severe medical conditions. Regenerative medicine is likely the future of treating numerous diseases. It is only a matter of time before many of these techniques are applied in clinical practice. These techniques provide prospects not only for repairing and modifying diseased cells but extend further to organs such as the kidneys, lungs, heart, and liver.

Nevertheless, multiple factors must be considered before administering such therapy to a potential patient. Primarily, the sex of both the donor and recipient, their age, and hormonal status should be taken into account to optimize the clinical outcome. Clinical studies on cardiac cell therapy have revealed significant sex-based differences in therapeutic responses. Meta-analyses of trials utilizing autologous BMSCs have shown that therapy in postmenopausal women resulted in significantly better outcomes compared to age-matched men. The mechanisms underlying this differential response are likely associated with epigenetic regulation (Thej et al., 2024). The aging process of stem cells also plays a critical role in therapeutic efficacy. Aged stem cells exhibit reduced proliferative and regenerative capacities. Clinical trials have demonstrated that advanced age of both the donor and the recipient is associated with decreased treatment efficacy (Fan et al., 2010; Ahmed et al., 2017; Chen et al., 2021). In experimental models, younger MSCs demonstrated superior therapeutic effects in infarcted rats, as evidenced by improved left ventricular (LV) ejection fraction and reduced LV volume, compared to older MSCs (Fan et al., 2010). In another study focused on bone regeneration, younger mice showed a more favorable response to MSC therapy than older animals. Another crucial factor is the influence of sex hormones, which play a vital role in modulating stem cell function. Research has demonstrated that estrogens exert a beneficial effect on the migration and proliferation of endothelial progenitor cells toward dysfunctional or ischemic tissues, including cardiac muscle (Ray et al., 2008).

To date, numerous scientific studies verified in clinical trials have demonstrated the potential for treating various diseases, including diabetes, liver cirrhosis, and heart or kidney failure (Aly, 2020; Petrosyan et al., 2022).

### 5.2 Type 1 diabetes

Hundreds of millions of people worldwide suffer from diabetes. This disease is associated with problems such as diabetic nephropathy, retinopathy, and neuropathy (Sneddon et al., 2018). There are two main types of diabetes: type 1 (T1D) and type 2 (T2D). T1D is characterized by the destruction of beta cells through autoimmune processes, leading to a complete deficiency of insulin, whereas T2D is characterized by insulin resistance, resulting in a relative deficiency of insulin supply (Memon and Abdelalim, 2020; Bourgeois et al., 2021). Patients with T1D require exogenous insulin to survive. However, injecting appropriate amounts at the right time is highly complex due to a wide range of factors that must be considered, highlighting the need for new therapeutic approaches (Hogrebe et al., 2023). Beta cells can develop from human ESCs and iPSCs (Verhoeff et al., 2021). A Phase 1 clinical trial involving a single patient yielded promising results: the patient with T1D achieved insulin independence 75 days after iPSC transplantation, and no abnormalities were observed after 1 year (Wang S. et al., 2024). MSCs are also being explored for T1D therapy (de Klerk and Hebrok, 2021). These cells possess remarkable properties. It is hypothesized that, apart from differentiating into islet cells, they can also protect them by secreting various cytokines and growth factors. Moreover, MSCs exhibit immunomodulatory effects, which appear to be the primary anti-diabetic mechanism (Cho et al., 2018). A study involving seven newly diagnosed T1D patients used MSC therapy derived from adipose tissue in combination with vitamin D supplementation for 6 months. The study demonstrated that this treatment was safe and beneficial for beta cell functionality and maintenance (Dantas et al., 2021). While these treatment methods hold promise, significantly more research is needed to determine their potential risks and long-term effects conclusively.

### 5.3 Heart failure

Heart failure (HF) is typically defined as a syndrome characterized by clinical features that include both structural and functional cardiac abnormalities, usually associated with reduced cardiac output and/or increased intracardiac pressure (Chandramouli et al., 2022). Approximately 64 million people worldwide suffer from HF (Shahim et al., 2023). It is evident that research into new therapeutic options is of paramount importance. Various categories of stem cells can be used in HF therapy (Müller et al., 2018). A clinical study conducted on female Wistar rats compared the therapeutic properties of MSCs and human ESCs. Although no definitive superiority of one therapy over the other was determined, co-transplantation of both cell types led to better LV functionality preservation compared to single-cell treatment. This result confirms that using multiple stem cell types may be more beneficial than a single type, though further clinical studies are required to validate this hypothesis (Puymirat et al., 2008). Another study involving patients suffering from severe ischemic heart failure with depressed LV systolic function demonstrated that BMSCs could play a crucial role in treating this condition. Among the thirty-two patients who completed the study, after two intracoronary infusions of BMSCs, there was an improvement in LV filling and an enhancement in NYHA functional class. However, no significant improvement was observed in LV ejection fraction (Diederichsen et al., 2010).

### 5.4 Liver cirrhosis

Liver cirrhosis is a disease that may result from multiple factors, including obesity, alcoholism, viral hepatitis B and C, autoimmune disorders, or excessive accumulation of certain elements such as iron or copper (Ginès et al., 2021). This chronic condition is characterized by fibrosis and regeneration of liver nodules, leading to portal hypertension and liver failure (Geong et al., 2019). Liver cirrhosis ranks 14th among the most common causes of death worldwide. It cannot be classified as a single disease entity due to the existence of multiple clinical stages (Tsochatzis et al., 2014). Common therapeutic approaches include eliminating etiological factors, administering anti-inflammatory and immunosuppressive pharmacotherapy, inhibiting activation and promoting apoptosis of hepatic stellate cells, and using hepatoprotective drugs, among others (Zhou et al., 2014). However, over time, some patients require liver transplantation, which is often challenging due to the limited availability of donors (Schuppan and Afdhal, 2008; Tsuchiya et al., 2019). A promising alternative is the use of MSCs, which possess anti-inflammatory and hepatoprotective properties, prevent hepatic stellate cell activation, and polarize macrophages into an anti-inflammatory phenotype, ultimately improving liver function (Tsuchiya et al., 2019). A study involving eight patients with liver cirrhosis (caused by different factors) demonstrated that MSCs injection into the peripheral or portal vein was well tolerated and led to improved liver functionality (Kharaziha et al., 2009). Another study applying autologous MSC therapy to a group of 25 patients with HCV-induced liver cirrhosis (12 of whom completed the study) reaffirmed its safety and efficacy. However, the authors emphasized that MSCs did not reach the liver in satisfactory quantities, suggesting that the observed clinical improvements were due to the immunomodulatory functions of MSCs, which facilitated the clearance of HCV RNA (Kantarcioʇlu et al., 2015).

### 5.5 Acute kidney injury

Acute kidney injury (AKI) is characterized by a sudden decline in glomerular filtration rate, resulting in the accumulation of metabolic waste products and ion homeostasis disturbances, often accompanied by increased serum creatinine levels (Hoste et al., 2018; Mercado et al., 2019; Ronco et al., 2019). AKI is associated with progressive renal dysfunction or continuous kidney impairment, which leads to irreversible nephron loss and may culminate in chronic kidney disease (CKD) (Kellum et al., 2021). AKI etiology can be classified as prerenal, renal, or postrenal (Mercado et al., 2019).

Complete renal function restoration is rarely achieved, making patients susceptible to complications that may result in mortality (Hoste et al., 2018). Thus, expanding medical knowledge on therapeutic options and their potential applications is crucial for improving the prognosis and quality of life of AKI patients. The use of human ESCs and iPSCs is being considered as a potential treatment due to their extraordinary regenerative potential, including for kidney tissues (Rota et al., 2019). However, there are currently insufficient studies to determine their utility in AKI treatment definitively. Studies conducted on mice have shown that MSCs can differentiate into renal tubular epithelium, demonstrating potential for kidney tissue regeneration. Moreover, MSCs exhibit the ability to secrete various cytokines, chemokines, and growth factors, further enhancing tissue regeneration and inducing cell proliferation (Yun and Lee, 2019). Unfortunately, human clinical studies using these treatment options remain scarce. Table 2 illustrates key findings.

**TABLE 2:** Summary of clinical trials regarding the therapeutic use of stem cells in various diseases.

	Study	Year	Methods	n	Aim	Outcomes
1	Wang et al.	2024	autologous transplantation of chemically iPSC beneath the abdominal anterior rectus sheath for T1D treatment	1	Evaluation of safety, tolerability, and efficacy of autologous chemically iPSC-islets as a therapeutic strategy for T1D	Patient achieved sustained insulin independence 75 days post-administration. Glycemic range increased from a baseline value of 43%–96% by month 4 after administration, and a decrease in glycated hemoglobin was noted
2	Dantas et al.	2021	A single dose of ASCs was administered in a peripheral upper arm vein. Patients started taking vitamine D_3_ oraly 2,000 UI 1 day after the infusion for 6 months	9	Evaluation of safety and efficacy of ASCs and daily vitamin D_3_ for 6 months in patients with recent-onset T1D	good glycemic control and low insulin requirements, without significant decline in β-cell function was reported in all patients, with little to none complications
3	Puymirat et al.	2008	Transplantation of MSCs, or ESCs, or both in an immunocompetent rat model of myocardial infarction	46	To determine whether MSCs and ESCs coinjection is associated with better cell engraftment and functional recovery	Combined aministration of ESCs and MSCs was associated with a greater functional benefit of LV, compared with controls, than either treatment alone
4	Diederichsen et al.	2010	Two intracoronary infusions of autologous BMSC every 4 months in patients with chronic ischemic HF with LV ejection fraction less than 40%	32	Evaluation of BMSC therapy on LV diastolic filling by measuring the ratio of transmitral flow velocity to early mitral annulus velocity, left atrial volume and plasma levels of N-terminal pro-brain natriuretic peptide	BMSC administrations had a beneficial effect on LV filling in patients with HF and this resulted in a sustained improvement in NYHA functional class
5	Kharaziha et al.	2009	Injection of 30–50 million MSCs into peripheral or the portal vein in patients with liver cirrhosis with different etiologies	8	Evaluation of feasibility, safety, and efficacy of using autologous MSCs as a treatment in patients with liver cirrhosis	Liver function improved as verified by a deacrease in Model for End-Stage Liver Disease score, prothrombin complex from international normalized ratio, serum creatinine, and an increase in serum albumin
6	Kantarcıoğlu et al.	2015	Patients received 1 × 106 autologous mesenchymal stem cells/kg injected into peripheral vein in patients with liver cirrhosis with different etiologies	25	Evaluation of autologous MSCs transplantation on liver tissue and liver chemistry	MSC transplantation was safe and feasible. Liver biopsy examinations revealed that MSC could not reach the liver in a adequate amount, but improvement in patients and clearance of HCV RNA was noted

## 6 Ethical considerations in regenerative medicine

There are several categories of stem cells such as hESCs, fetal stem cells and ASCs. ASCs, unlike the others, do not present ethical dilemmas (Stoltz et al., 2015). Human ESCs are sourced from pre-implantation embryos (Sampogna et al., 2015). At the current moment the only way for them to be acquired is through the destruction of the human embryo, so this present a serious ethical issue (de Miguel-Beriain, 2015). This aspect raises numerous controversies among scientists worldwide, as they are confronted with two fundamental moral principles. The first emphasizes the prevention and alleviation of suffering in individuals affected by disease. The second upholds the duty to respect human life and acknowledge its immense value. The Genetic Science Learning Center has summarized the most critical and challenging questions regarding stem cell research. Does life begin at the moment of fertilization, during embryo implantation in the uterus, or only after birth? Is a human embryo equivalent in value to a human child? Does a human embryo possess any rights? Does saving numerous human lives justify the destruction of a single embryo that could serve as the source for developing a cure? (The Stem Cell Debate: Is it Over?, 2025). The last question closely relates to the so-called trolley problem, first described by Philippa Foot (1967) and later expanded upon by Judith Jarvis Thomson (1976). The thought experiment presents a runaway trolley hurtling down the tracks. On its current path, five people are tied to the tracks, but an individual can pull a lever to divert the trolley onto another track, where only one person is present (Bruers and Braeckman, 2014). What should be done in such a situation? No universally satisfactory answer has been found to this and the previous questions, and it is unlikely that one ever will be. However, there is a category of stem cells that raises fewer controversies on the moral front. This refers to iPSCs, which were discovered and developed by Kazutoshi Takahashi and Shinya Yamanaka. This groundbreaking discovery has made it possible to generate pluripotent cells from differentiated adult human cells. iPSCs represent an extremely promising source of cells with functions similar to human ESCs, which can be obtained from the same patient without the need to destroy an embryo (Mao and Mooney, 2015; Cossu et al., 2018). Although iPSCs do not require the destruction of an embryo, they are still not free from ethical dilemmas. As mentioned earlier, iPSCs have the ability to differentiate into any cell type. This property primarily enables their use in the creation of gametes (both sperm and oocytes). Such applications may contribute to the treatment of infertility and, theoretically, open the door to human reproductive cloning. However, these cells should not be used for such purposes. Therefore, informed consent should be obtained from patients participating in therapies involving stem cells (Zheng, 2016; Volarevic et al., 2018; Moradi et al., 2019). The Nuremberg Code and the Declaration of Helsinki have, for decades, explicitly stated that unethical research involving human subjects is not permitted (Moradi et al., 2019). However, fully applying these principles to stem cell research and therapies is not entirely straightforward, which has led to the development of numerous guidelines and regulatory bodies across different regions of the world. In the United States, the regulatory authority overseeing stem cell research is the Food and Drug Administration (FDA), while guidelines are outlined in various documents, such as the National Institutes of Health Guidelines for Research Using Human Stem Cells (2009), which govern research on human ESCs and the practical application of iPSCs. Additionally, Institutional (or Embryonic) Stem Cell Research Oversight Committees (ISCROs/ESCROs) play a crucial role in maintaining integrity, ethical compliance, and public trust in stem cell research (Caulfield et al., 2014; Moradi et al., 2019; Charitos et al., 2021). In Europe, the equivalent of the FDA is the European Medicines Agency (EMA), which also establishes relevant guidelines. Furthermore, in the United Kingdom, the Gene Therapy Advisory Committee supervises clinical research related to stem cell and gene therapies. In Germany, the Central Ethics Committee for Stem Cell Research performs a similar function, overseeing the potential use and importation of stem cells. Despite the existence of multiple regulatory bodies, guidelines, and legal provisions worldwide, one fundamental principle unites all nations: the use of stem cells or any other methods for human reproductive cloning is strictly prohibited, unethical, and punishable by law.

Regardless of the above, we decided to examine the issue of ART, closely related to the topic under discussion, from a multicultural perspective. Aware of the differences in normative frameworks among various communities, we undertook a brief analysis concerning morality and the acceptance of conscience. Imagining the psychological pain and suffering of an infertile couple longing for offspring, assisted reproduction may appear to be a ray of light in the darkness, offering peace and hope. However, the problem is multidimensional—one must consider not only the desire to achieve conception but also the associated religious and ethical aspects. According to traditionalist perspectives, ART disrupts the intimate relationship inherent to natural conception by introducing artificial, human-engineered processes. Some publications even compare children conceived through ART to “laboratory products,” thereby presenting the practice as fundamentally opposed to the traditional family model (Negrut and Pop, 2022). Furthermore, the debate extends beyond the use of donor gametes to include the cryopreservation of gametes and embryos, as well as the possibility of sex selection. Depending on religious beliefs, it is assumed that the embryo acquires a soul at different stages, which significantly affects the ethical interpretation of these procedures. For example, in Islam, it is believed that the soul is imparted after 40 days; according to Protestant traditions, after 14 days; while the Catholic doctrine holds that life—and thus the possession of a soul—begins at the moment of fertilization. The deliberations of participants in the panel organized by the Ethics Committee of the Association for Fertility and Reproductive Health in Nigeria conveyed a general message that decisions regarding the use of ART are highly dependent on individual circumstances. In the sphere of bioethics, it is inappropriate to categorize actions as strictly right or wrong, as moral evaluation must be contextual and culturally sensitive (Bamgbopa et al., 2018).

## 7 Advanced regenerative technologies: challenges, constraints, and future directions–a discussion

The advancement of medical embryology, in conjunction with emerging technologies, marks the beginning of a new chapter in the future of regenerative medicine. Through an interdisciplinary approach that integrates embryology, tissue engineering, biotechnology, and AI, it is now possible to develop innovative therapeutic strategies based on stem cells. Their application offers vast therapeutic potential in clinical practice, including targeted therapies, gene therapy, and cell-based treatments. In recent years, progress in this field has enabled the implementation of the first effective gene therapies (Ponomarev et al., 2023). Another notable development involves the use of 3D bioprinting, cell-based therapies, stem cells, and biomaterials for the repair of damaged tissues and organs, as well as the fabrication of transplantable tissue constructs (Shang et al., 2022). The ultimate vision is to achieve affordable, non-toxic therapies that do not rely on long-term immunosuppressive drug administration (Edgar et al., 2020). The aim of this discussion is to analyse the potential of these novel technologies, explore their current limitations, and assess future perspectives regarding their application in regenerative medicine.

### 7.1 Modern technologies in regenerative medicine

Gene therapy represents a rapidly evolving branch of regenerative medicine. It is an advanced method that modifies the genome by introducing genetic material via a vector, with the aim of replacing a defective gene (Gonçalves and Paiva, 2017). Two main approaches are distinguished: *in vivo*, where the vector is directly administered into the patient’s body, and *ex vivo*, where cells are harvested from the patient, genetically modified outside the body, and then reintroduced (Arabi et al., 2022). The development of gene-editing technologies such as the CRISPR/Cas9 system has enabled precise modification of genetic material within stem cells (Razavi et al., 2024). The ability to alter DNA in human embryos represents a promising future in preventing congenital genetic disorders at the earliest stages of cellular development (Alnasser, 2021). One of the key achievements in gene therapy is the clinical use of Onasemnogene Abeparvovec, commercially known as Zolgensma, for the treatment of spinal muscular atrophy. In this disorder, a mutation in the *SMN1* gene leads to the loss of alpha motor neurons, which control muscle function, resulting in progressive atrophy. The therapy involves the delivery of a functional copy of the *SMN1* gene directly into the body, enabling production of essential proteins required for the survival and proper functioning of motor neurons (Ogbonmide et al., 2023; Shchaslyvyi et al., 2023). Another emerging application of gene therapy is in oncology, where genes can be introduced into tumors, immune cells are genetically modified to enhance anti-tumor responses, and cancer vaccines are being developed (Akbulut, 2020; Sayed et al., 2022). Notably, the integration of artificial intelligence (AI) in gene therapy could significantly enhance therapeutic outcomes. Through machine learning (ML), genome analysis can be optimized to select the most appropriate gene-editing tools (Asadi Sarabi et al., 2024). One example of ML application is the CRISPR-GEM algorithm. It enables prediction of optimal gene-editing targets, permitting precise selection of genes to achieve the intended therapeutic effect (Graham et al., 2024). A critical aspect of regeneration is the regulation of gene expression, as regenerative processes often recapitulate patterns observed during embryogenesis (Goldman and Poss, 2020). Numerous studies highlight the importance of understanding signaling pathways involved in gene expression related to organogenesis and tissue repair. Stem cells, with their unique capacity for self-renewal and differentiation into specialized cell types, play a fundamental role in regenerative medicine. The use of stem cells for the cultivation of organoids opens new possibilities for organ development studies, disease modeling, and the design of personalized therapies. Organoids, miniature structures allow for drug testing without the need for animal models (Margiana et al., 2022; Silva-Pedrosa et al., 2023; Park et al., 2024). One example of AI application in organoid culture is its ability to streamline the development of this technology. AI customizes personalized culture conditions, performs quality control, and carries out a detailed analysis of the collected data (Bai et al., 2023). Cell therapy is another widely employed technique in regenerative medicine. It involves the transplantation of autologous or allogeneic cells to enhance the regenerative potential of tissues with limited repair capacity. A notable example is the use of autologous chondrocytes for knee cartilage repair, where healthy cartilage cells are harvested from the patient, expanded *in vitro*, and reintroduced into the damaged site (El-Kadiry et al., 2021; Makarczyk, 2023). 3D bioprinting, a key innovation in tissue engineering, offers an alternative to traditional transplants. This technology enables the creation of biologically functional tissue structures by layering bio-ink composed of biomaterials compatible with the human body (Kantaros, 2022). Owing to its precision in cell placement, it is possible to produce tissues that mimic native human tissues in both form and function (Zhang et al., 2021). Bioprinting also holds great potential in bone tissue regeneration, facilitating fracture healing through the creation of scaffolds that accelerate the repair process and promote functional recovery (Maresca et al., 2023). Thanks to FRESH technology (Freeform Reversible Embedding of Suspended Hydrogels), an *in vitro* full-scale model of the human heart was successfully printed. This model reproduces the anatomical structure but exhibits no biological activity (Mirdamadi et al., 2020). Scientists studying *in vivo* bioprinting obtained tissue resembling an ear, in which histopathological examination revealed the presence of chondrocytes. This represents a significant step toward a novel technique for auricular reconstruction (Chen et al., 2020).

### 7.2 Global disparities in regulation

Now we will examine the differences in regulations concerning stem cells and ATMPs (advanced therapy medicinal products) To regulate clinical trials involving ATMPs, the EU introduced Regulation 1,394/2007, which streamlined the marketing-authorization procedure. ATMPs were defined as gene therapy products, somatic-cell therapy products, and tissue-engineered products (Priesner and Hildebrandt, 2022; Schuessler-Lenz et al., 2023). In the European Union, the law rigorously addresses safety and ethical matters in human-embryo research. In contrast, the United States generally adopts a more liberal approach to stem-cell research (Song S. J. et al., 2024) In 2017 in the United States, the FDA introduced the Regenerative Medicine Advanced Therapy (RMAT) designation, aimed at streamlining drug development and potentially accelerating the approval of these therapies (Vaggelas and Seimetz, 2019). Other programs in the United States include Fast Track and Breakthrough Therapy, which facilitate the implementation of new drugs (Iglesias-Lopez et al., 2020). An analysis of regulatory pathways in highly developed countries such as the United States, Japan, South Korea, and EU nations revealed the existence of accelerated pathways that enable more efficient approval of new therapies (Qiu et al., 2020). Between 2010 and 2020, only 15 ATMPs were approved in the EU, and 14 in the United States of America. Despite these advances, regulatory barriers obstruct the availability of these products. Collaboration among centres worldwide is essential to streamline ATMP regulation and approval (Warreth and Harris, 2020). In low- and middle-income countries, regulatory frameworks are the least developed. In most of these nations, there is a lack of guidelines aligned with their economic circumstances. Many may be unable to afford the development of advanced technologies such as gene therapies (Olayemi et al., 2017; Cornetta et al., 2022). As a result of legal gaps worldwide, the concept of cell tourism has emerged. In low-income countries, clinics offering stem-cell therapies of questionable efficacy have arisen. These clinics attract travellers from across the globe (Lyons et al., 2022). Legal oversight of these therapies and the introduction of regulations to limit this precedent are essential.

### 7.3 Challenges and limitations of contemporary regenerative technologies

Despite significant technological advances, several technical limitations persist. In the case of cell-based therapies, one major challenge is cellular heterogeneity, which hampers the standardization of treatments across patient populations. Research has demonstrated that the application of such therapies may increase the risk of oncogenic transformation, for example, into hepatocellular carcinoma, highlighting the necessity for further safety evaluations (Petrosyan et al., 2022). Another concern is the elevated risk of rejection of transplanted stem cells due to the limited expression of tissue-specific antigens, which leads the recipient’s immune system to recognize these cells as foreign (Wang J. et al., 2024). The bioprinting of large organs, such as the heart, remains a considerable challenge due to the limited oxygen diffusion capacity through bioprinted tissue. The current resolution of 3D printing technologies does not allow for the fabrication of capillary networks, which are essential for the effective delivery of oxygen and nutrients to cells (Ramadan and Zourob, 2020). Additionally, the high costs associated with acquiring, maintaining, and operating 3D printers present a significant barrier to the clinical implementation of this technique (Saini et al., 2021). Another ongoing challenge is the development of suitable bioinks used in 3D bioprinting. These materials must be biodegradable, non-toxic, and compatible with the recipient’s cellular matrix (Thakur et al., 2023). Addressing issues such as host immune response, lack of vascularization, and ensuring the long-term stability of applied solutions is critical for the advancement of regenerative technologies (Yuan et al., 2025). Although gene therapy holds enormous therapeutic potential, numerous barriers hinder its widespread application, particularly those related to treatment costs. One example is the single-dose therapy for spinal muscular atrophy, which currently costs approximately two million euros per dose—a price inaccessible to many patients globally (Nuijten, 2022).

**FIGURE 3:**
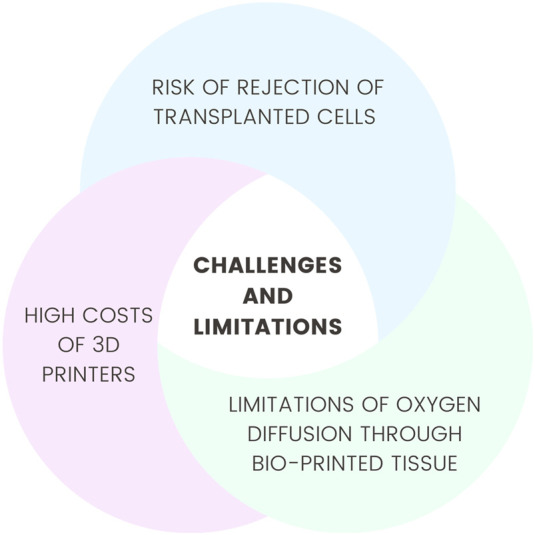
Challenges and limitations.

In conclusion, the synergy between medical embryology and regenerative medicine offers promising avenues for the treatment of various diseases. Continued interdisciplinary collaboration, alongside the advancement of current technologies, is essential for revolutionizing regenerative medicine and improving patient care. Future research should prioritize reducing production costs, increasing accessibility, and addressing immunological challenges and the durability of therapeutic approaches. Figure 3 shows challenges and limitations in this topic.

## 8 Conclusions

The unification of medical embryology with regenerative medicine marks a groundbreaking milestone in contemporary biomedical sciences. This work has demonstrated that embryological mechanisms of stem cells, signaling pathways, and microenvironmental interactions constitute a foundational basis for the development of regenerative therapies, particularly due to their remarkable potential in treating previously terminal conditions such as diabetes, heart failure, liver cirrhosis, and kidney failure. Likewise, organoids, which emerge from the convergence of embryology, biotechnology, and tissue engineering, appear to be an extraordinary tool for disease modeling. Their ability to restore organ function under *ex vivo* conditions offers a socially acceptable alternative to animal experimentation and provides a platform for drug testing and therapy selection. However, several limitations still remain, including inadequate vascularization, matrix substrate variability, and size constraints, all of which clearly point to the need for further improvements, methodological refinement, and standardization. Modern technologies such as gene therapy, 3D bioprinting, and AI also represent a major leap forward in advancing precision medicine. Nonetheless, these approaches face considerable challenges, including immune incompatibility, ethical concerns, and the high cost of innovative treatments. Therefore, this field requires vigilant regulatory oversight and the establishment of universal guidelines to support responsible and sustainable innovation.

In summary, the synthesis of embryology and regenerative medicine heralds a new era in medical science, potentially enabling organ repair, disease reversal, and a general enhancement of human health and quality of life. Achieving this future, however, will depend on sustained interdisciplinary research, regulatory governance, and effective analysis of clinical outcomes to ensure the successful translation of laboratory discoveries into clinical practice.
